# Pectin: A Bioactive Food Polysaccharide with Cancer Preventive Potential

**DOI:** 10.3390/molecules27217405

**Published:** 2022-10-31

**Authors:** Talha Bin Emran, Fahadul Islam, Saikat Mitra, Shyamjit Paul, Nikhil Nath, Zidan Khan, Rajib Das, Deepak Chandran, Rohit Sharma, Clara Mariana Gonçalves Lima, Ahmed Abdullah Al Awadh, Ibrahim Abdullah Almazni, Abdulaziz Hassan Alhasaniah, Raquel P. F. Guiné

**Affiliations:** 1 Department of Pharmacy, BGC Trust University Bangladesh, Chittagong 4381, Bangladesh; 2 Department of Pharmacy, Faculty of Allied Health Sciences, Daffodil International University, Dhaka 1207, Bangladesh; 3 Department of Pharmacy, Faculty of Pharmacy, University of Dhaka, Dhaka 1000, Bangladesh; 4 Department of Pharmacy, International Islamic University Chittagong, Chittagong 4318, Bangladesh; 5 Department of Veterinary Sciences and Animal Husbandry, Amrita School of Agricultural Sciences, Amrita Vishwa Vidyapeetham University, Coimbatore 642109, Tamil Nadu, India; 6 Department of Rasa Shastra and Bhaishajya Kalpana, Faculty of Ayurveda, Institute of Medical Sciences, Banaras Hindu University, Varanasi 221005, Uttar Pradesh, India; 7 Department of Food Science, Federal University of Lavras, Lavras 37200-900, Brazil; 8 Department of Clinical Laboratory Sciences, Faculty of Applied Medical Sciences, Najran University, P.O. Box 1988, Najran 61441, Saudi Arabia; 9 CERNAS Research Centre, Department of Food Industry, Polytechnic Institute of Viseu, 3504-510 Viseu, Portugal

**Keywords:** pectin, food polysaccharide, cancer, modified citrus pectin, apoptosis

## Abstract

Pectin is an acidic heteropolysaccharide found in the cell walls and the primary and middle lamella of land plants. To be authorized as a food additive, industrial pectins must meet strict guidelines set forth by the Food and Agricultural Organization and must contain at least 65% polygalacturonic acid to achieve the E440 level. Fruit pectin derived from oranges or apples is commonly used in the food industry to gel or thicken foods and to stabilize acid-based milk beverages. It is a naturally occurring component and can be ingested by dietary consumption of fruit and vegetables. Preventing long-term chronic diseases like diabetes and heart disease is an important role of dietary carbohydrates. Colon and breast cancer are among the diseases for which data suggest that modified pectin (MP), specifically modified citrus pectin (MCP), has beneficial effects on the development and spread of malignancies, in addition to its benefits as a soluble dietary fiber. Cellular and animal studies and human clinical trials have provided corroborating data. Although pectin has many diverse functional qualities, this review focuses on various modifications used to develop MP and its benefits for cancer prevention, bioavailability, clinical trials, and toxicity studies. This review concludes that pectin has anti-cancer characteristics that have been found to inhibit tumor development and proliferation in a wide variety of cancer cells. Nevertheless, further clinical and basic research is required to confirm the chemopreventive or therapeutic role of specific dietary carbohydrate molecules.

## 1. Introduction

It is feasible to remove pectin from the cell walls of non-graminaceous plants, which may subsequently be used as a thickening agent for culinary and medicinal purposes. For commercial purposes, it is standard practice to recover pectin from plant material that has been left behind after the juice or sugar has been collected from the plant. Industrially, pectins must adhere to strict guidelines to be approved as a food additive by the Food and Agricultural Organization, which stipulates that they must contain at least 65% polygalacturonic acid. Fruit pectin derived from oranges or apples is often used in the food sector to gel or thicken goods and to stabilize acid-based milk beverages. Although sugar beet pectin extracts are ineffective gelling agents in the laboratory, the potential use of sugar beet pectin extracts as emulsifiers and coagulants in the industrial setting is quite intriguing [1,2].

The vast majority of the possible health benefits associated with pectin are thought to be due to its role as a soluble dietary fiber. Lipogenesis, or the production of fatty acids within the body, is induced by pectin fermentation, which occurs in the colon and is responsible for producing fatty acids. Pectin is poorly digested in the small intestine but fermented by bacteria in the colon. It becomes a rich source of short-chain fatty acids, including acetate, propionate, and butyrate [1,2]. Consuming soluble fiber has been found to reduce blood levels of low-density lipoprotein (LDL) cholesterol, reducing the risk of heart disease. Generally speaking, this function, together with pectin’s ability to bind to bile salt micelles, is thought to be connected to pectin’s ability to prevent the micelles from being reabsorbed back into the circulation [3]. Although the existence of binding has been confirmed, its exact nature remains incompletely understood. However, there has been a number of tried-and-true strategies proposed to account for this particular kind of binding [4]. Several studies have hypothesized that pectin may be used in anti-constipation and anti-diarrhea preparations because of its superior moisturizing properties compared to other types of fiber [5].

Modified pectin (MP), specifically modified citrus pectin (MCP), is gaining popularity as evidence accumulates of its high efficacy in reducing the development and spread of malignancies, such as colon and breast cancer. The modification of pectin as part of a newly discovered chemical process releases, or predisposes the release of, fragments of the pectin molecule during intestinal digestion [6]. During digestion, these fragments bind to and block the activity of the pro-metastatic regulatory protein galectin 3 (Gal-3), inducing tumor progression. This finding is supported by data from various settings, including cellular and animal studies and human clinical trials.

This review will explore various structural and functional properties of pectin and the outcomes of the modification processes that have been used to generate MP. It will also cover the evidence that these modified materials are bioactive as well as the possible outcomes related to this characteristic [7], and discuss how pectin fragments may preferentially bind to oncogene *Gal-3* and its possible repercussions on oncogenesis and cancer prevention. It is expected that many other dietary carbohydrates with immunomodulatory and anticancer effects, such as β-glucans, will have behaviors similar to pectin, and the absorption mechanisms for pectin itself will be examined and discussed in more detail [8]. The purpose of this review is to describe the role of pectin in cancer prevention in depth, progressing from preclinical findings to prospective therapeutic applications. The pharmacokinetic, toxicological, and bioavailability properties of pectin are also covered in this review.

## 2. Methodology

**Figure 1:**
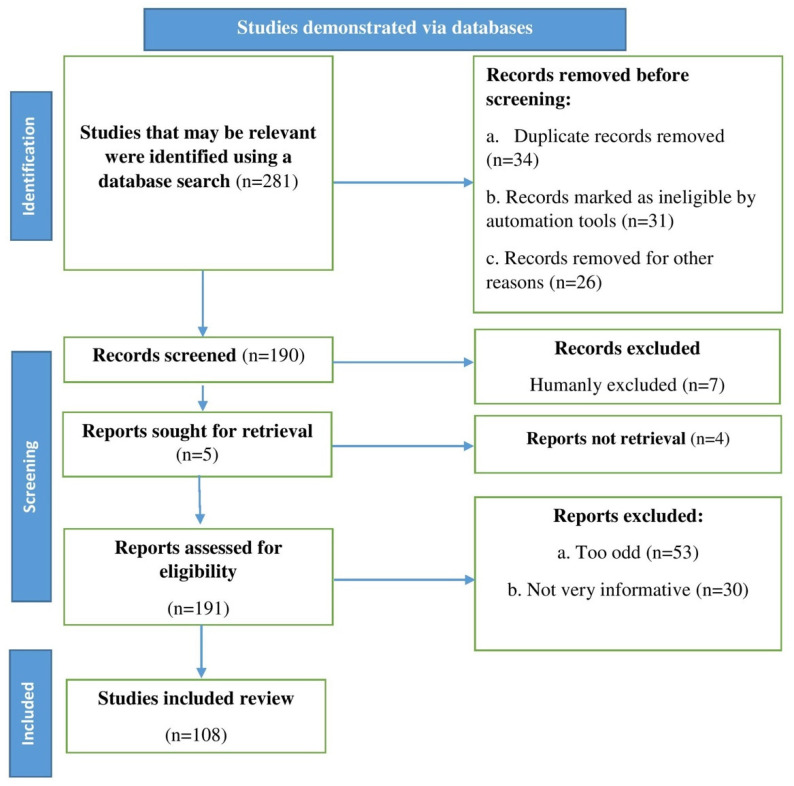
Stages involved in selecting published data for inclusion in this study are depicted in a flow chart. Key: n, number of literatures.

We conducted a literature search and gathered recent and relevant articles from various databases, including Scopus, Science Direct, Elsevier, PubMed, and Web of Science. We used the following search terms: pectin, anticancer properties, bioavailability, pharmacokinetics, and clinical trials. Research reports, review articles, and original research articles written in English were selected and evaluated. We also reviewed their references and included them where appropriate. We used an algorithm built according to Page et al. [9] to manage all of the processes involved in identifying the relevant evidence for this study (Figure 1). The exclusion criteria for other reasons were not appropriate.

## 3. Anticancer Properties of Pectin

### 3.1. Breast Cancer

Nearly 1.7 million new cases of breast cancer arise annually worldwide, accounting for 25.2% of all cancer cases (WHO). Approximately 15% of breast cancer patients die following diagnosis, second only to lung cancer [10,11]. Complex, molecular breast cancer occurs when gene mutations cause abnormal cell growth and proliferation [12]. In vitro and in vivo studies have shown that pectin-derived chemicals slow cell development and promote apoptosis [13]. The synergistic and additive effects of MCP have been studied in human breast cancer cells. Polybotanical compounds BreastDefend (BD) or ProstaCaid (PC) used in combination with MCP reduced the invasive potential of highly metastatic human breast cancer MDA-MB-231 cells. MCP may also prevent the development of human breast cancer in mice by reducing angiogenesis, a crucial mechanism for tumor growth. The α-galactosidase binding protein Gal-3 is one potential mediator of MCP’s inhibition of breast and prostate cancer cell adhesion, migration, and invasion [7]. MCP inhibits urokinase-type plasminogen activator (uPA) synthesis in breast and prostate cancer cells, and the uPA receptor (uPAR) regulates cell adhesion, migration, and invasion [14]. In addition to activating macrophages, CP decreased activator protein 1 (AP-1) and NF-κB signaling but increased LPS/Toll-like receptor 4 (TLR-4) signaling [15]. In another study, CP and apple pectin (AP) inhibited MDA-MB-231, MCF-7, and T47D breast cancer cells, finding that CP and AP inhibited cancer cells in the S and G1 or G2/M phases of the cell cycle. Similarly, the expression of *Gal-3*, a lectin implicated in cell adhesion, cell cycle, and death, was reduced by CP and AP. In addition, oxidative and strand-break DNA damage was observed in MDA-MB-231 cells, slowing proliferation [16].

**Figure 2:**
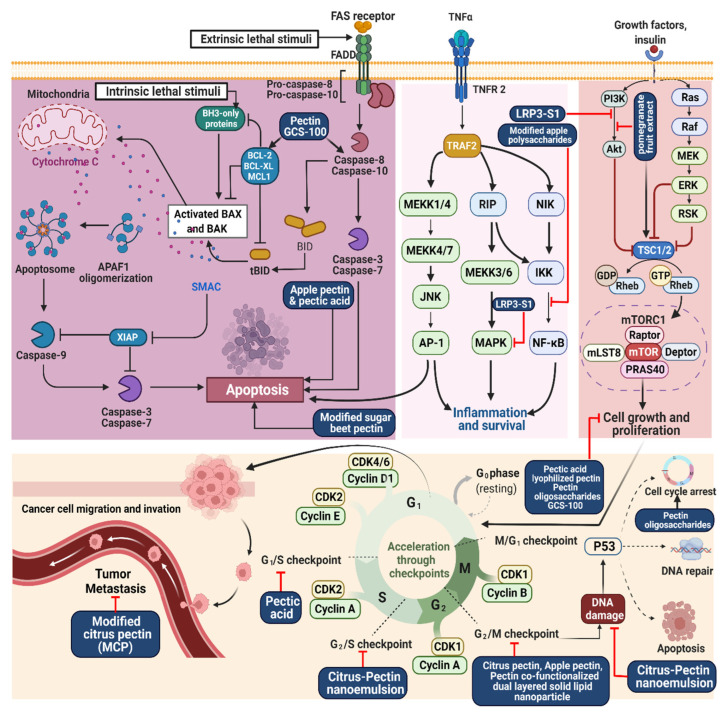
Illustration representing the site of action of pectin and associated pectins in cancer treatment.

CP with Zataria multiflora essential oil (ZEO) stimulated the mitochondrial intrinsic apoptotic pathway by producing reactive oxygen species (ROS) and inhibiting matrix metalloproteinases (MMPs) in MDA-MB-231, T47D, and MCF-7, L929 human breast cancer cells. DNA damage and CP/ZEO nanoemulsions (NEs) interference induced cell death [17]. AP suppressed the growth of 4T1 breast cancer cells by inducing apoptosis in vitro [18]. MCF-7 and MDA-MB-231 cancer cell lines were also explored by using pectin gold nanoparticles mediated (P-GNPs), finding that they induce apoptosis based on flow cytometry and morphological changes in both cell lines, increasing the sub-G1 population indicative of apoptosis [19] (Figure 2).

The toxicity of human lymphocytes against MCF-7 breast cancer cells was assessed by using a pectin guar gum zinc oxide nanocomposite, finding it increased IFN-γ, interleukin 2 (IL-2), and TNF-α cytokine release, CD3, CD8, and CD56 expression, and cancer cell mortality [20].

### 3.2. Gastric Cancer

Pectin has also shown promise in preventing stomach cancer. The use of low-molecular-weight CP (LCP) as a chemopreventative in gastric cancer has been explored with the AGS cell line, showing that LCP with 5-fluorouracil (5-FU) reduced cyclin A (*CCNA1*) and B1 (*CCNB1*) expression to a greater extent than LCP alone. In addition, LCP inhibited intestinal cancer cell proliferation and metastasis by inhibiting B-cell lymphoma-extra-large (*Bcl-xL*) and cyclin B expression and epithelial–mesenchymal transition (EMT) [21]. Another study explored the ability of MCP to prevent the generation of organized emboli and reduce tumor development [22]. Moreover, MCP reduced the number of active metastases [23], perhaps reflecting its small size and galactose-rich side chains, enabling it to adhere to tumor cell surface galectins, preventing them from attaching to host cell surfaces.

### 3.3. Colon Cancer

The increasing mortality rate of colon cancer makes it a growing health concern. It is the third most frequent cancer in both sexes worldwide, currently accounting for almost 10% of all cancer cases and anticipated to reach 60% by 2030, reflecting over 2.2 million cases. To further understand the function of pectin in colon cancer, several in vitro studies have been reported with various human colon cancer cell lines. In addition, an in vivo study has assessed the hypothesis that a fiber pectin (FP) diet reduces signaling through the cyclooxygenase (COX) and Wnt/β-catenin pathways, resulting in reduced levels of the antiapoptotic transcription factor peroxisome proliferator-activated receptor delta (PPARD). This study used 40 Sprague–Dawley weanling male rats divided equally into two groups. One group received only azoxymethane (AOM) injections, while the other received AOM injections with irradiation at 1 gray (Gy) of 1 GeV/nucleon iron ions [24]. Rats were fed an FP diet to determine how it affected the COX and lysyl oxidase (LOX) pathways. They found the FP diet to inhibit both the COX (microsomal prostaglandin E synthase-2 [mPGES-2] and prostaglandin E2 [PGE2]) and Wnt/β-catenin pathways but increased PPARD levels. Another study explored the effects of pectin supplementation during the promotive stage of dimethylhydrazine (DMH)-induced carcinogenesis, finding a substantial reduction in the occurrence of colon cancer but no change in the number of tumors per rat [25].

A further study used Wistar DMH-treated rats to explore how AP formulations affect neoplastic damage and proliferative marker dysregulation, finding a substantial beneficial effect on colon carcinogenesis through the regulation of physiological indicators, oxidative stress, inflammatory markers, and hemodynamic alterations [26]. The anti-cancer activities of phenolics from apple trash have also been studied in HT29, HT115, and CaCo-2 cell lines as in vitro models of colon carcinogenesis. They found a significant decrease in HT115 cell invasion when apple phenolics were used to prevent DNA damage, a process associated with tumor initiation. In addition, hydrogen peroxide (H_2_O_2_)-induced damage was significantly reduced in HT29 cells, and CaCo-2 cell barrier function was increased, a process associated with reduced tumor promotion and metastatic potential [27].

Several studies have indicated that pectin induces apoptosis in human colon cancer cells (Table 1). The suppression of HT-29 colon cancer cell proliferation and cell cycle progression was explored by using potato (rhamnogalacturonan) pectin [28]. They found potato rhamnogalacturonan I domain-rich pectin (p-RG-I) reduced HT-29 cell growth and caused G2/M cell cycle arrest characterized by decreased *CCNB1* and cyclin-dependent kinase 1 (*CDK1*) expression. Real-time quantitative reverse transcription PCR (qRT-PCR) was used to detect mRNA levels of cell cycle arrest protein [28], and the 3-(4,5-dimethylthiazol-2-yl)-2,5-diphenyltetrazolium bromide (MTT) test and flow cytometry to assess proliferation and cell cycle distribution. They found p-RG-I to have a dose-dependent effect on cell growth inhibition based on the MTT test. Cell proliferation was decreased to 90%, 79%, 67%, and 55% of control after 72 h of treatment with p-RG-I at concentrations of 0.6, 1, 25, 2, and 5 mg/mL, respectively [29]. Another study found ultrasonic modified sweet potato pectin (SSPP) induced apoptosis-like cell death in colon cancer (HT-29) cell lines in a dose-dependent manner in vitro. Moreover, the anticancer activity of 400 W SSPP was much higher than that of 1 or 3 mg/mL of heat-fragmented CP in BCaP-37 and HT-29 cell lines, which has been shown to induce over 50% cell death in HepG2 and A549 cell lines [30]. Moreover, both 0.5 mg/mL 200 W and 400 W SSPP induced a significant percentage of apoptotic cells [31], increasing caspase 3 (CASP3) activity, the last step of apoptosis, and phosphatidylserine (PS) release, indicative of intermediate apoptotic phases [32].

In vivo studies on the anti-metastatic properties of pectin are currently being performed (Table 1). One study found that MCP treatment reduced liver metastases in the colon of 75 Balb mice administered MCP at doses of 0, 0, 1, 2, 5, and 10% (*w*/*v*). Enzyme-linked immunosorbent assay (ELISA), gene expression microarray, and immunohistochemical results showed that MCP significantly inhibited the growth and spread of colon cancer in mouse spleens. The lower incidence of liver metastases and smaller tumor volumes with high MCP concentrations show that it may inhibit colon cancer growth and metastasis in a dose-dependent manner. However, ELISA and immunohistochemistry showed no differences in Gal-3 levels or expression in liver metastatic cancer cells despite MCP reducing liver metastasis [33].

Another study found that adding soluble CUR to pectin and skimmed milk powder nanoparticles (DL-SLN) increased cytotoxicity by increasing levels of tumor suppressor protein p53 and cysteine acid proteases such as cyclin-dependent kinases 2 (Cdk2), 4 (Cdk4), and 6 (Cdk6), and reducing the expression of cyclin D1 (CCND1). In addition, a cell cycle study has shown DL-SLN-treated SW480 cells during the G2/M phase. In addition, both PC and PectaSol-C (SolC) have been shown to dramatically reduce the viability of the HCT 116 and CaCo-2 colon cancer cell lines. Moreover, the proliferation of colon cancer cells was found to be inhibited by pectins extracted from ginseng and potatoes. However, no synergic effect between SolC and 7-ethyl-10-hydroxycamptothecin (SN-38) in lowering colon cancer cell viability was observed. Studies have investigated the ability of pectin to induce apoptosis to better understand its antioxidant properties, finding that PC increased apoptosis whereas SolC had no effect, activating CASP3 in the sub-G1 cellular fraction but not general apoptosis. However, PC was found to enhance ROS levels in colon cancer cells but not SolC. In addition, a concentration of 5 nM SN-38 slightly elevated ROS levels when administered alone. Moreover, LPS-treated HCT 116 cells produced considerably more interleukin 6 (IL-6) and cyclooxygenase 2 (COX-2) after pectin treatment [34,35].

The flora in the colons of CRC patients differs from that of healthy individuals. It has been shown that PC and SolC have a significant effect on *E. coli* adhesion to colorectal cancer cells by reducing their ability to stick to them. The influence of pectin on the expression of genes associated with mitochondrial fusion and fission in human colorectal HT29 cancer cells has been explored. Specific mitochondrial proteins maintain a dynamic balance between mitochondrial fission and fusion. Both mitofusin 1 (Mfn-1) and 2 (Mfn-2) were more abundant in HT29 cells that had been cultured with pectin than those that had not, indicating that pectin inhibits mitochondrial fission. Moreover, it was found that pectin significantly decreased CCNB1 expression in HT-29 cells, which is essential for progression from G2 to M, and enhanced pectin transfer from the cytoplasm to the nucleus caused growth cessation [36].

### 3.4. Pancreatic Cancer

It is estimated that only 6% of patients with pancreatic ductal adenocarcinoma live beyond five years without recurrence [34]. Many studies have explored the effects of pectin on pancreatic cell lines. The pectin-like polysaccharide RP02-1 derived from *Polygala tenuifolia* was found to have an inhibitory effect on pancreatic cancer cell development in vitro and in vivo by promoting apoptosis and reducing autophagy in pancreatic cancer cells. Treatment of pancreatic cancer cell line HPDE6-C7 with RP02-1 increased CASP3 activity and the BCL2-associated X apoptosis regulator (Bax) to BCL2 apoptosis regulator (Bcl-2) ratio but reduced chromatin condensation. RP02-1 treatment also interfered with autophagy markers Beclin 1 (BECN1), autophagy-related 5 (ATG5), and autophagy-related ubiquitin-like modifier LC3 B (LC3B), indicating that it suppressed autophagy in BxPC-3 pancreatic cells (Table 1) [35]. In addition, the anti-pancreatic ductal carcinoma activity of pectin derived from *Lycium ruthenicum* Murr (LRP3-S1) was evaluated in AsPC-1, PANC-1, and BxPC-3 pancreatic cancer cell lines, finding it inhibited their development in a dose-dependent manner by altering signaling through the mitogen-activated protein kinase (MAPK) and focal adhesion kinase (FAK)/protein kinase B (AKT)/glycogen synthase kinase-3 (GSK-3) pathways [36].

### 3.5. Hepatocellular Cancer

Hepatocellular carcinoma (HCC) is the fifth-most common cancer worldwide, with an estimated 500,000 new cases and 600,000 deaths annually [37,38]. The cytotoxic effects of pectin-capped gold nanoparticles (PEC-AuNPs) and doxorubicin (DOX) and their combination (DOX-PEC-AuNPs) on HepG2 and HeLa cells have been compared. The cytotoxicity of DOX-PEC-AuNPs was found to be dose-dependent (Figure 2), with the viability of HepG2 and HeLa cells decreasing with increasing dosage. The concentration of pure DOX was 4.11 mg/mL, and the concentration of DOX-PEC-AuNPs was 0.74 mg/mL in HepG2 cells. In addition, the DOX and DOX-PEC-AuNPs half-maximal inhibitory concentration (IC_50_) values in HeLa cells were 3.88 and 3.27 mg/mL, respectively. However, the IC_50_ value for DOX-PEC-AuNPs in HepG2 cells expressing the asialoglycoprotein receptor (ASGPR) was significantly different from that for DOX, indicating their greater effectiveness in killing cancer cells [39]. Another study has explored the anticancer effects of pectin-based nanoparticles for treating HCC in vivo and in vitro by using the MTT assay with the HepG2 and A549 cancer cell lines (Table 1). However, although pectin-based nanoparticles were biocompatible, they appeared to increase cancer cell proliferation. In addition, 5-FU nanoparticles were found to kill HepG2 cells more efficiently than free 5-FU with the ASGPR enabling delivery to target cancer cells [40].

### 3.6. Bladder Cancer

Urinary bladder cancer (UBC) was the sixth-most frequent malignancy among males worldwide in 2012 [41]. With an anticipated 60,490 new cases in 2017, it is now the fourth-most frequent cancer among males in the US [42]. Several in vitro studies have evaluated the effect of pectin on bladder cancer (Table 1). MCP was found to inhibit bladder tumor formation by downregulating *Gal-3* in human UBC cell lines T24 and J82. Moreover, MCP inhibited UBC tumor development by causing cell cycle arrest and death in vitro and in vivo. In high-quality UBC samples, MCP reduced the expression of *Gal-3*, which has functions related to cell proliferation, survival, adhesion, and metastasis [43,44] and suggests a low overall survival rate. Akt and MAPK signaling pathways are inactivated by MCP therapy but not MAPK itself. G2/M cell cycle arrest and death in UBC cells were observed when Gal-3 was targeted with MCP [45]. In addition, pectic oligosaccharides (POS) reduced the proliferation, migration, and invasion of 5637 and T24 bladder cancer cells in vitro, increasing apoptosis and inducing S phase cell cycle arrest. Moreover, POS also increased cell motility, apoptosis, and expression of cell cycle-related proteins and decreased expression of GLI family zinc finger 1 (*Gli1*) and sonic hedgehog (*Shh*) and Gli1 nucleocytoplasmic translocation [46].

### 3.7. Prostate Cancer

Oral prevention of prostate cancer metastases is possible. Oral MCP therapy appears to interfere with the natural ligand recognition of tumor cell surface galectins required for successful colony growth. In addition, early-stage metastasis may be slowed by MCP, decreasing tumor cell emboli formation and cancer cell endothelial contact [22]. Moreover, MCP was found to kill androgen-dependent and non-androgen-dependent prostate cancer cells in vitro and significantly reduce MAPK signaling associated with cell growth, survival, and death [47].

The effect of pectin on prostate cancer cell lines has been studied in vitro. Studies found that MCP decreased PCa cell survival and simultaneously increased their susceptibility to infrared radiation (IR) in a dose-dependent manner. However, a slight decrease in the G0/G1 phase was caused by MCP, leading to substantial cell arrest in G2. In addition, MCP was found to increase the production of the pro-apoptotic protein Bax and cleavage of the CASP3 precursor. Moreover, apoptosis suppression, angiogenesis, adhesion, motility, and invasion were significantly decreased with the combined use of MCP and IR, whereas MCP alone had no effect. It has been proposed that Gal-3 downregulation and inhibition of its anti-apoptotic effect cause the enhanced radiosensitivity and cell death observed with combined MCP and IR treatment. Further evidence suggests that the combination of MCP-mediated Gal-3 inhibition and the functions of Gal-3 in tissue remodeling and fibrosis may reduce the adverse effects of radiation treatment [48].

PectaSol acts synergistically with DOX to treat DU-145 and LNCaP prostate cancer cell lines by reducing viability and proliferation. Moreover, the combined use of PectaSol and DOX reduced their IC_50_ values by 1.3- and 1.5-fold, respectively, in LNCaP cells. Unlike PectaSol therapy, DOX causes G2/M arrest. In addition, PectaSol appears to provide a synergistic effect with DOX, causing increased apoptosis (sub-G1 arrest) in human PCa DU-145 cells and G2/M arrest in LNCaP cells. Moreover, *p53* expression was unaffected by their combined use, whereas *p27* expression was reduced [49].

### 3.8. Ovarian Cancer

An early in vitro study on the cytotoxic effect of sulforaphane (SFN) in ovarian cancer found Gal-3-enhanced SKOV-3 cell proliferation, collagen adhesion, and anti-apoptotic collagen action. Its inhibition of *CCND1* expression caused G1 arrest by reducing cyclin E2 (CCNE2) and D2 (CCND2), and CDK6 while increasing the cell cycle inhibitor p21^Cip^, with a dose-dependent increase in SKOV-3 cell proliferation observed. Moreover, the anti-apoptotic effect of Gal-3 on SKOV-3 cells was neutralized by Bcl-2 activation and CASP3 inhibition [50]. Another study found Gal-3 to induce signal transducer and activator of transcription 3 (STAT3) activation and paclitaxel synergy in the presence of Pectasol-C MCP. Numerous studies have connected STAT3 constitutive activation to chemoresistance in ovarian cancer cells [51,52,53]. The injection of exogenous Gal-3 resulted in increased STAT3 phosphorylation (pSTAT3) in ovarian cancer cells. In addition, a study examining how PectaSol-C MCP (PectMCP) with or without paclitaxel (PTX) affected pSTAT3 levels in SKOV-3 mice engineered to produce three different mammalian cell lines found a reduction in AKT activity in the presence of PectMCP, suggesting that it sensitizes SKOV-3 cells to PTX via altered integrin expression (Table 1) [54].

### 3.9. Leukemia

Activation marker tests and functional natural killer (NK) cell activity assays have been used to assess the immunostimulatory capabilities of MCP in blood cultures. MCP was found to increase cytolysis of leukemic cells, which is regulated by cytotoxic chemicals, activating receptors, and inhibitory receptors. MCP caused a T helper 1 (Th1) cell polarization in response to activated T cytotoxic and NK cells except at low doses (<0.08%), consistent with prior preclinical studies, and may be effective for immune system modulation [55]. Gal-3 activates the PI3K activation pathway and the protein kinase C (PKC) and ROS pathways shared by activation and apoptosis. Interestingly, ginseng pectins were found to inhibit the ROS pathway but not the PI3K pathway, inhibiting Gal-3-induced T-cell death but not activation [56]. An antiproliferative effect of olive pectin extracts was reported in CaCo-2 and THP-1 cell lines at doses between 1 and 10 mg/mL. Moreover, GAL-3, a lectin involved in tumor formation and immune cell regulation, was found to reduce red blood cell aggregation. Finally, lactate dehydrogenase (LDH) and CASP3 tests showed that pectin-rich extracts trigger apoptosis in THP-1 cells [57].

### 3.10. Myeloma

Studies on the effect of pectin in myeloma therapy are ongoing. Alkali-soluble pectin reduces immunoglobulin E (IgE) production in human myeloma cell line U266 in a study examining the effects of water- (WP), hexametaphosphate- (HXP), acid- (HP), and alkali-soluble (OHP) pectin solutions. However, whereas OP altered cell viability, WP, HXP, and HP did not. Moreover, pectin increased the proportion of IL-2 receptor (IL-2R)-positive cells in mesenteric lymph node (MLN) lymphocytes, indicating that its immunoglobulin modifying effect was mediated by activated Th1 cells [58].

The ability of a new Gal-3 antagonist, GCS-100, to induce myeloma cell death by modulating MCL1 apoptosis regulator BCL2 family member (MCL-1), phorbol-12-myristate-13-acetate-induced protein 1 (NOXA), and the cell cycle was assessed in the U266 and RPMI 8226 cell lines. This study found it to decrease *MCL-1*, *BCL-XL*, *NOXA*, and *p21^Cip1^* expression in myeloma cells, leading to concurrent decreases in cyclin E and *CCND2*. The rate of sub-G1 phase cells increased in S phase cells, indicating apoptosis, which may be mediated by a decrease in signal transduction, as indicated by the downregulation of AKT, IκB, p65 NF-κB, and IκB kinase (IKK) after 24 h [59]. GCS-100 also reduced bortezomib resistance and increased dexamethasone-induced apoptosis in multiple myeloma cells via caspase 8 (CASP8)/CASP3 PARP pathway activation. However, inhibiting CASP8 but not caspase 9 (CASP9) decreases GCS-100-induced cell death. Therefore, it is likely that GCS-100 first affects cell surface carbohydrate-binding proteins because cell surface death receptors are known to trigger CASP8 via Fas-associated via death domain (FADD) [60,61]. In addition, GCS-100, which targets cell-surface lectins, significantly suppressed the proliferation and migration of multiple myeloma cells in the presence of bone marrow stromal cells (BMSCs), suggesting that it impairs multiple myeloma-to-BMSC connections, preventing tumor cell proliferation and survival. However, although GCS-100 alone did not influence *GAL-3* expression, its use in combination with dexamethasone did [62].

### 3.11. Skin Cancer

Several recent studies have shown that pectin has anti-melanoma effects [63]. Pectin was first studied for its potential to reduce the risk of skin cancer in a laboratory setting. CP is a natural complex carbohydrate shown to affect Gal-3-related characteristics in murine melanoma cells. In addition, although MCP inhibited laminin-induced cell adhesion, CP did not affect cell binding or spreading to laminin, a ligand for surface-expressed soluble Gal-3 molecules, in B16-F1 cells. However, 0.5% MCP had no influence on cell survival or proliferation in vitro. Therefore, its inhibitory effect cannot be attributed to cytotoxicity. Moreover, whereas MCP treatment reduced homotypic aggregates, CP treatment enhanced them, perhaps due to branched carbohydrate polymers acting as cross-linker bridges between neighboring cells [64]. ELISA shows recombinant Gal-3 binds MCP in a dose-dependent manner [65]. A separate study explored pectin’s cytotoxic and anti-proliferative effects in HaCaT cells. MCP had an IC_50_ of about 500 µg/mL, which was cytotoxic, and dose-dependent for both pectin and MCP [66]. The antiproliferative effect of Gal-3 in mice treated with MCP was enhanced by rhamnogalacturonan (RG-I), which is present in its structure [67].

### 3.12. Brain Cancer

Many studies have shown the efficacy of pectin treatment on brain cancer to be favorable. The cytotoxic effects of *Campomanesia xanthocarpa* pectins on human glioblastoma U251-MG and T98 G cells have been studied [68]. They found that treatment with crude pectin extract (GW) and soluble purified pectin (GWP-FP-S) at 25–400 µg/mL caused substantial alterations in U251-MG cell morphology relative to a vehicle-treated control group after 48 h based on the crystal violet test. Another study using the MTT assay confirmed that high doses of GW and GWP-FP-S (100–400 µg/mL) were cytotoxic to all cell types, regardless of origin, and that lower amounts of pectin produced a different response. Moreover, the 2’-7’dichlorofluorescin diacetate (DCFH-DA) staining showed substantial increases in intracellular ROS levels following treatment with 100–400 µg/mL GW and 25–400 µg/mL GWP-FP-S compared to untreated U251-MG cells [68].

### 3.13. Lung Cancer

Lung cancer was predicted to be the leading cause of death in cancer fatalities in the United States in 2016 [69,70]. Many in vitro studies have used various human colon cancer cell lines to explore the effect of pectin on lung cancer. One study found A549 lung adenocarcinoma cells to be killed by a nanocomposite of pectin (Pec), guar gum (gg), and zinc oxide (Pec-gg-ZnO) at concentrations of 25–200 µg/mL based on the MTT assay. The IC_50_ values of Pec-gg-ZnO showed that it was bioactive in the A549 cell line. Incubation with Pec and gg biopolymers and Pec-gg-ZnO enhanced apoptosis in A549 lung cells at the Sub-G1 phase. The DCFH-DA staining in Pec- and gg-treated cells increased 2- and 1.8-fold, respectively, when their ROS production was measured by intracellular H_2_O_2_ levels. Moreover, normal DNA fragmentation was observed in A549 cells treated with Pec-gg-ZnO but not Pec and gg after 24 h [71].

Studies have shown in situ that gold pectin-modified iron oxide (Fe_3_O_4_) nanoparticles inhibited the growth of lung cancer LC-2/ad cells and human umbilical vein endothelial cells (HUVECs) after 48 h based on the MTT assay. Moreover, they had IC_50_ values of 137, 143, and 252 µg/mL against PC-14, HLC-1, and LC-2/ad cells, respectively [72]. PFE was found to inhibit phosphorylation of JNK1/2, which are involved in cell proliferation and transformation, and p38 in cells, reducing tumor growth, proliferation, and transformation (Table 1) [73].

**Table 1:** Preclinical experimental evidences on the use of pectin and modified pectins in cancer.

Cancer Type	Drug	Dose	Study Model	Outcomes	Ref.
Breast cancer	PectaSol-C modified citrus pectin (MCP)	MCP (0.25–1.0 mg/mL)	In vitro (human breast cancer cells)	↓ Breast cancer cell migration and suppress adhesion of breast cancer cells	[15]
	Pectic acid	(0, 0.1, 0.1, 0.5, 1% *w*/*v*)	In vitro (4T1 breast cancer cells)	Induce apoptosis, ↓ cell growth ↓ cell attachment, fragmented chromatin, blocked the sub-G1 phase	[18]
	Pectin-mediated gold nanoparticles (p-GNPs)	(2, 4, 6, 8 and 10 µg/mL)	In vitro (human breast adenocarcinoma cell lines)	↓ Cell viabilities ↑ Sub-G1 population	[19]
	Citrus-pectin nanoemulsion	5,10, and 65.5 μg/mL of CP/ZEO	In vitro (human breast cell line and normal fibroblasts cell)	↑ Reactive Oxygen Species (ROS) ↑ mitochondrial membrane potential (MMP) loss ↑ DNA damage ↑ G2 and S-phase arrest	[17]
	Modified citrus pectin (MCP)	1% (*w*/*v*) MCP	In vivo (athymic mice)	↓ Tumor growth ↓ Angiogenesis ↓ Spontaneous metastasis	[74]
	Citrus pectin or apple pectin	1% (*w*/*v*) Cp or Ap	In vitro (human breast cell line and normal fibroblasts cell)	Suppressed the viability in MDA-MB-231, MCF-7 and T47D human Breast cancer cells, ↓ mRNA expression of galectin-3	[16]
	Pectin-guar gum-zinc oxide (PEC-GG-ZnO)	(25 µg/mL to 200 µg/mL)	In vitro (breast cancer cell lines, MCF-7)	Enhancing cytotoxicity towards lung ↑ effector: target ratios from 2.5:1 to 20:1 ↑ Cancer cell death	[20]
Gastric cancer	Low-molecular-weight citrus pectin (LCP)	(0.625 to 10.0 mg/mL)	In vitro (AGS gastric cancer cell-line)	↓ Cell viabilities ↓ Cyclin B1 expression ↓ Galectin-3 (GAL-3) expression	[23]
	Pectic-oligosaccharide	(10, 20 and 30 µg/mL)	In vitro (AGS human gastric carcinoma cells)	↓ Galectin-3 activity ↓ Growth of AGS cells Inducing apoptosis	[75]
	pH-modified citrus pectin (MCP)	low-dose MCP (0.8 mg/mL) high-dose MCP (1.6 mg/mL)	In vivo (mice)	↓ Tumor size	[21]
Colon cancer	Modified citrus pectin (MCP)	0.0%, 0.0%, 1.0%, 2.5% and 5.0% (*w*/*v*)	In vivo (Balb/c mice)	↓ Liver metastases ↑ Serum galectin-3	[33]
	Lyophilized pectin	1 to 10 mg mL^−1^	In vitro (Caco-2 cells)	Antiproliferative effect, ↓ Agglutination of red blood cells by galectin-3	[57]
	Apple extract	Apple extracts (0.01%, 0.02%, 0.05% and 0.1%)	In vitro (HT29, HT115, and CaCo-2 cell lines)	Protection against DNA damage, inhibit invasion	[27]
	Sweet potato pectin (SPP)	0.0025 g/mL of SPP	In vitro (HT-29 cells)	↑ Galacturonic acid (GalA), arabinose and galactose content, ↓cell proliferation, induced apoptosis	[31]
	Pectin	FP diets contained 3.5 g of corn oil per 100 g of diet	In vivo (Sprague–Dawley rats)	Enhanced colonocyte apoptosis suppressing of PPARd and PGE2 elevating of PGE3	[24]
	Pectin	5%, 10% pectin	In vivo (Sprague–Dawley rats)	Suppressing colon carcinogenesis	[25]
	Pectin-encrusted gold nanocomposites	DMH + PA-PGNPs (PA equivalent to 2 mg/kg/day, oral)	In vivo (Albino rats of Wistar strain)	Suppression of colon carcinogenesis, dysregulating of proliferation markers	[26]
	High and low methoxy pectins (HP and LP)	0.01–1.0 mg/mL	In vitro (Caco-2 cells)	Concentration-dependent effect on inhibiting of Caco-2 cells proliferation	[76]
	Pectin oligosaccharides	(0.1 mg/mL to 1 mg/mL)	In vitro (colon cancer HT-29 cell line	↓ Proliferation ↑ Cytotoxicity ↓ Cell viability	[77]
	Modified sugar beet pectin	0.2, 0.5 or 1.0 mg/mL	In vitro (HT29 and DLD1 colon cancer cells)	Inducing apoptosis Cell cycle arrest Reducing proliferation	[78]
	Rhamnogalacturonan I domain-rich pectin	0 or 5 mg/mL	In vitro (colon cancer HT-29 cell line)	Inhibiting proliferation Significant G2/M cell cycle arrest. Downregulate cyclin B-1 and cyclin dependent kinase 1 expression	[29]
Pancreatic cancer	Pectin-like polysaccharide named RP02-1	(0 µM, 4.31 µM, 8.62 µM)	In vitro (pancreatic cell line HPDE6-C7)	↓ Cancer cell proliferation, migration and colony formation, induced pancreatic cancer cells apoptosis, suppressed autophagy	[35]
	LRP3-S1	(0, 4.36, 8.71 μM)	In vitro (pancreatic cancer cell lines AsPC-1, BxPC-3, PANC-1)	Attenuated invasion ability, downregulated protein expression of p-FAK, p-AKT, p-GSK-3β and p-p38 MAP kinase	[36]
	Pectin	0–1000 μg/mL	In vitro (BxPC-3 and PANC-1 cells)	Inhibit cell growth	[79]
Colorectal cancer	Thiolated pectin–doxorubicin (DOX) conjugate	(Equivalent to 0.15 mg/kg DOX)	In vivo (BALB/c mice)	Inhibited the growth of all cell lines, primary tumor growth and suppressed tumor metastases	[80]
	Apple pectin	(0.05–0.5 mg/mL with or without 5 nM SN-38)	In vitro (human colon cancer cell lines)	↓ Viability of HCT 116 and Caco-2 inducing apoptosis, ↑ Intracellular ROS production, ↑ Cytotoxic and proapoptotic effect of irinotecan	[81]
	Citrus pectin and modified citrus pectin	(20%)	In vivo (Fischer 344 rats)	Rise to a tumorigenesis prevention, ↓ pH in caecum lumen and increase in acetate and lactic acid levels	[82]
	Pectin co-functionalized dual layered solid lipid nanoparticle	Pectin solution (2%)	In vivo (zebrafish model)	Arresting G2/M phase, improving the oral bioavailability of curcumin (CMN)	[83]
	Heat-treated Helianthus annuus L. pectin (HT-HAP), alkali-inactivated HT-HAP	150, 300, 110 or 220 mg/kg body weight	In vivo (female Balb/c mice)	Induced apoptosis, reduced tumor growth	[84]
	Pectin	200 µmol/L pectin.	In vitro (HT-29 cells)	↓ Expression of dynamin-related protein-1. ↑ Expression of the mitochondrial fusion-associated proteins mitofusin-1 and 2. Blockade of G2/M transition. ↑ Expression of p53 protein	[85]
	Pectin	(0.25, 0.5 and 1 mg/mL)	In vitro (HT-29 cells)	Inhibited adhesion, invasion, proliferation and anchrogen-independent growth	[86]
	Modified apple polysaccharides	(1.0–0.01 mg/mL)	In vitro (CRC cell lines, HT-29 and SW620)	Reduced LPS-induced NF-κB expression Suppressed LPS-induced migration and invasiveness	[87]
Hepatocellular cancer	5-FU loaded pectin-based nanoparticles (5-FU-NPs)	0.5 to 0.006 mM for 5-FU and 5- FU-NPs	In vitro (HepG2 and A549 cells)	Exhibited size-induced prolonged circulation as well as ASGP receptor-mediated targeting ability to cancer cell lines	[40]
	Pectin-deoxycholic acid	Citrus pectin (1.54 g)	In vitro (HepG2 cells)	↑ Cytotoxicity, ↓ Relative migration of HepG2 cells, ↑ micelles cellular uptake	[88]
	Pectin-capped gold nanoparticles (PEC-AuNPs)	100 mL of 0.03% Pectin solution	In vitro (human Caucasian hepatocyte cells)	Greater potency in killing, proving a promising carrier for anticancer drug	[39]
Bladder cancer	Modified citrus pectin (MCP)	0.125 to 2%, (*w*/*v*)	In vitro (T24 and J82 human UBC cells)	↓ Cell proliferation, ↓ galectin-3 Inactivation of Akt signaling pathway, ↓ tumor growth	[45]
	Pectin oligosaccharide (POS)	0–30 µg/mL	In vitro (SV-HUC-1 cells)	Promoted the apoptosis of bladder cancer cells, activated the Hedgehog pathway	[46]
Prostate cancer	Modified citrus pectin	0.01–1.0% (*w*/*v*)	In vivo (Rats)	Inhibited MAT-LyLu cell adhesion	[22]
	PectaSol-C modified citrus pectin (MCP)	0.1%, 1.0%	In vitro (LNCaP and PC3 cells)	Inhibited MAP kinase activation, ↑ expression level of its downstream target Bim, a pro-apoptotic protein, and induced the cleavage of Caspase-3 in PC3 and CASP1, ↓ cell proliferation and apoptosis	[47]
	Modified citrus pectin	0.3%	In vitro (human prostate cancer cells)	↑ Cisplatin-induced apoptosis of PC3 cells, ↑ calpain activation	[89]
	Modified citrus pectin	25 mg/mL	In vitro (human prostate carcinoma cells)	↓ Gal-3, cleavage of the precursor of caspase-3, ↑ expression of the pro-apoptotic protein Bax, ↓ DNA repair pathways, poly-ADP-ribose polymerase	[48]
	Fractionated pectin powder	(0.01–3 mg/mL)	In vitro (Prostate cancer cell lines, LNCaP and LNCaP C4-2)	Induced apoptosis	[90]
	Modified citrus pectin (Pectasol)	PectaSol (0.5, 1, 3 and 5 mg/mL),	In vitro (Human PCa DU-145 and LNCaP cells)	↑ Sub-G1 arrest, G2/M arrest ↑ p53, p27 and Bcl-2 expression Inducing cell death through apoptosis and cell growth arrest	[49]
Ovarian cancer	Modified citrus pectin (Pect-MCP)	Pect-MCP (0.025, 0.05, 0.1%)	In vitro (human ovarian cancer SKOV-3 cells)	↑ Cell proliferation, ↓ caspase-3 activity, ↑ substrate-dependent adhesion in the presence of rhGal-3	[50]
	PectaSol-C modified citrus pectin (Pect-MCP)	Pect-MCP (0.025%)	In vitro (human ovarian cancer SKOV-3 cells)	↓ Expression of downstream target HIF-1α ↓ integrin mRNA levels ↓ AKT activity	[54]
Cervical cancer	Pectin, guar gum and zinc oxide nanocomposite	(25, 50, 100 and 200 μg/mL)	In vitro (Cervical adenocarcinoma (HeLa) cell lines)	Induced mitochondrial depolarization, reactive oxygen species generation, caspase-3 and Poly (ADP-ribose) polymerase 1 (PARP1) activation resulting in DNA fragmentation.	[71]
Leukemia	Modified citrus pectin (MCP)	(0–800 μg/mL)	In vitro (blood samples and normal lymphocytes)	Activated T-cytotoxic cells B-cell, and NK-cells	[55]
	Ginseng pectins	(0.1, 0.5, 2 mg/mL	In vitro (Jurkat cells (human leukemia T-cell line))	↓ ROS/ERK pathway, ↑ T-cell proliferation and IL-2 expression ↓ Tumor growth by 45%, ↓ Gal-3-induced T-cell apoptosis	[56]
	Lyophilized pectin	1 to 10 mg mL^−1^	In vitro (THP-1 cells)	Activation of caspase-3 in THP-1 cells, triggered apoptosis	[57]
	Pectic oligosaccharides	(5%)	In vivo (male BALB/c mice)	↓ Metabolic alterations ↑ Acetate in the caecal content. Counteracted the induction of markers controlling β-oxidation	[91]
Myeloma	GCS-100	GCS-100 (0–800 µg/mL)	In vitro Myeloma cell lines U266 and RPMI 8226	Induced inhibition of proliferation, accumulation of cells in sub-G1 and G1 phases, and apoptosis with activation of both caspase-8, upregulating cell-cycle inhibitor p21Cip1, reduction in signal transduction	[59]
	Alkali-soluble pectin	5%	In vitro (human myeloma cell line)	Suppressed IgE production	[58]
	GCS-100	350 or 700 µg/mL 220 or 500 µg/mL	In vitro (multiple myeloma cell lines MM.1S, MM.1R, RPMI-8226, LR-5, U266, and DOX-40)	Inhibited multiple myeloma cell growth, induced apoptosis, activated caspase-8 and caspase-3	[62]
Skin cancer	pH-modified citrus pectin (MCP)	0.5% CP or 0.5% MCP.	In vitro	Inhibited anchorage-independent growth, Inhibited cells adhesion to laminin and asialofetuin	[65]
	Modified citrus pectin (MCP) or citrus pectin	0.05% CP or 0.05% MCP.	In vitro (B16-F1 melanoma cells)	↓ B16-F1 experimental metastasis	[64]
	Pectin or MCP	(1–750 µg/mL)	In vitro (HaCaT cell line)	Exhibited a stronger cytotoxic and anti-proliferative effect	[67]
Brain cancer	Pectin extract (GW)	10–400 μg mL^−1^ of GW	In vitro (U251-MG and T98 G human glioblastoma cell lines)	Induced cytotoxicity, ↑ cellular ROS levels	[68]
	Modified pectin	50μL stock 3% solution	In vitro (C6 glioma cells)	↓ Metabolism of C6 glioma cells, ↓ Cell viability	[92]
Lung cancer	Pectin-guar gum-zinc oxide (PEC-GG-ZnO)	(25 µg/mL to 200 µg/mL)	In vitro (lung cancer cell lines, A549)	Enhanced cytotoxicity towards lung ↑ effector: target ratios from 2.5:1 to 20:1, ↑ cancer cell death	[20]
	Pectin-modified magnetic nanoparticles (Fe3O4/Pectin/Au)	(0–1000 μg/mL)	In vitro (human lung cancer cell lines)	Lowest IC50 values	[72]
	Pectin, guar gum and zinc oxide nanocomposite	(25, 50, 100 and 200 μg/mL)	In vitro (lung adenocarcinoma (A549))	Induced mitochondrial depolarization, reactive oxygen species generation, caspase-3 and Poly (ADP-ribose) polymerase 1 (PARP1) activation resulting in DNA fragmentation	[71]
	Pomegranate fruit extract (PFE)	(0.1 and 0.2%, *w*/*v*)	In vitro (lung adenocarcinoma (A549))	Inhibition of tumor growth, ↓protein expressions of cyclins D1, D2 and E ↓ cyclin-dependent kinase (cdk) 2, cdk4 and cdk6 expression. Inhibition phosphorylation of MAPK proteins, PI3K, Akt, NF-kB and IKKa, (v) degradation	[73]
	Pectin-PVP based curcumin particulates	CP3 (2.5, 25 and 250 µg/mL)	In vitro (A549 cells are adenocarcinomic human alveolar basal epithelial cells)	Enhancement in anti-tumor potential	[93]

## 4. Clinical Trials with Pectin in Cancer

Prostate cancer patients undergoing targeted therapy such as radical prostatectomy, radiation, or cryosurgery were studied to determine whether Pecta-Sols MCP was tolerated and effective. In the study by Guess et al. [94] 800 mg Pecta-Sols powder-filled capsules were administered to each participant in the trial, with a total dosage of 18 capsules per day (14.4 g), to assess whether the prostate-specific antigen (PSA) doubling time (PSADT) of prostate cancer patients changed after taking MCP. Seven of the 10 evaluable instances showed a statistically significant rise in PSADT in which each patient functioned as their own control. Increasing the time required for the PSA level to double would, if maintained, indicate that cancer development would be delayed, potentially leading to a longer life expectancy (Table 2) [94,95].

The tolerability, therapeutic benefit, and anticancer efficacy of MCP were studied in 49 advanced solid tumor patients to enhance their quality of life and reduce palliative symptoms, such as pain, cachexia, asthenia, malaise, psychological distress, nausea, and vomiting. Overall, six of the 29 patients who took MCP for two cycles showed a clinically beneficial response with stable or improved life quality. Moreover, stable disease (SD) was found in 11 of the 49 patients (22.5%) with an ITT basis after two cycles, whereas an SD was found in 6 of the 49 patients (12.3%) with an ITT basis for more than 24 weeks. PSA levels decreased by 50% in prostate cancer patients after 16 weeks of therapy, resulting in a substantial improvement in clinical benefit, quality of life, and pain reduction [98].

## 5. Toxicological Profile

An erythrocyte hemolysis assay was used to determine the cytotoxicity of pectin cerium oxide nanoparticles (Pe-CeO_2_NPs), finding for the first time that when Pe-CeO_2_NPs are detected in their natural habitat, they are biocompatible with living creatures [99]. Conversely, when the dosage of Pe-CeO2NPs was increased from 0.05 to 8.00 mg/mL, both the amount (0.55–8.31%) and rate (0.55–8.31%) of hemolysis increased. A 4 mg/mL concentration of Pe-CeO_2_NPs was found to cause 4.55% hemolysis and was determined to be the appropriate concentration for human consumption based on the permitted hemolysis limit established for biocompatibility studies of materials and biomaterials [99]. It was found that a zebrafish toxicity model was effective in this study.

Subsequently, zebrafish embryos were used to explore the toxicity of pectin gold nanoparticles (Pe-AuNPs) [100]. They concluded that the toxicity did not affect zebrafish once they were in the growth stage since no malformations were identified in the eggs examined under a microscope. At Pe-AuNPs dosages between 200–1000 ng/mL, there was no change in the survival rate of zebrafish. Therefore, Pe-AuNPs are very well suited for biological and pharmacological delivery applications [101]. In addition, a study evaluated the acute and subacute toxicity of Pe-AuNPs in Sprague–Dawley rats, finding no signs of mortality, organ damage, or abnormalities [102], concluding that Pe-AuNPs are completely safe when administered orally at single dosages of 5–10 mg/kg [103]. Moreover, when rats were given the same dose every day for four weeks, subacute toxicity tests found no abnormal changes or severe side effects. Therefore, pe-AuNPs are safe at subacute concentrations of exposure based on the findings of in vivo acute and subacute toxicity studies, even at high dosages [104].

Fibroblast NHDF cells have been treated with pectin silver nanoparticles (Pe-AgNPs; 0.001 M Ag in 1% pectin) to assess their cell cytotoxicity compared to pure pectin and media containing fetal bovine serum [105]. They found that using a 1:20 dilution of Pe-AgNPs provided a viability percentage of 120–140%, whereas pure pectin had a vitality percentage of 105–110%, similar to the viability percentage of media containing fetal bovine serum. Based on their findings, they concluded that Pe-AgNPs are not harmful and can increase the vitality of NHDF cells [106,107,108].

## 6. Concluding Remarks and Future Directions

The cytotoxic effects of pectin provide it with anti-cancer properties, which have been shown to inhibit tumor growth and proliferation. MCPs inhibit almost all cancer cells via inhibition of the MAPK signal pathway. The high monogalacturonic acid content of PectaSol-C may make it easier to absorb and disperse than other PectaSols. LRP3-S1 inhibited the MAPK and FAK/AKT/GSK-3 signaling pathways in pancreatic cancer cell lines BxPC-3, PANC-1, and AsPC-1. Colon cancer cells invade the liver through galectins, carbohydrate-based recognition proteins. The uPA receptor (uPAR) controls cell adhesion, migration, and invasion, and MCP inhibits the urokinase-type plasminogen activator (uPA) production in breast and prostate cancer cells. CP boosted LPS/Toll-like receptor 4 (TLR-4) signaling while decreasing AP-1 and NF-B signaling and activating macrophages in addition. In another study, CP and apple pectin (AP) suppressed MDA-MB-231, MCF-7, and T47D breast cancer cells. This inhibition occurred in the S and G1 or G2/M stages of the cell cycle, the researchers found. By preventing the production of B-cell lymphoma-extra-large (Bcl-xL), cyclin B, and the epithelial-mesenchymal transition, LCP reduced the growth and spread of intestinal cancer cells in gastric cancer (EMT). Another research looked into how MCP may inhibit the formation of ordered emboli and lessen the growth of tumors. The capacity of tumor cells to attach to the endothelium and basement membrane of blood arteries is required for liver metastasis. Therefore, galectins may be required in different phases of metastasis. Finally, MCPs reduced in vitro cell survival independent of androgen dependence. CASP3 activation and MAPK inhibition may restrict cell growth and enhance apoptosis in the presence of MCPs. Therefore, further clinical and basic research is required to establish the role of specific dietary carbohydrate molecules as chemopreventive or therapeutic agents.

## Figures and Tables

**Table 2:** Clinical evidences on the use of pectin and modified pectins in cancer.

Study Design	Study Population	No. of Patients/Control Subjects	Status	Findings	References
Randomized, crossover, comparison study	Italy	69	Completed	Decrease in pain intensity of >33% was observed in 16, 102, and 159 treated episodes at T5, T10, and T20, respectively	[96]
Nonrandomized phase II pilot study	United states	13	Completed	Significant (*p*-value < 0.05) increase in PSADT after taking MCP for 12 months.	[94]
Single-center, open label, trial, phase-II	Israel	60	Completed	Decreased/stable PSA in 58% (*n* = 34), or improvement of PSADT in 75%.	[95]
Randomized, double-blind, crossover study	United states	114	Completed	Improved pain intensity (PI) scores as early as 5 min (*p* < 0.05); pain intensity difference (PID) from 10 min (*p* < 0.01); and pain relief (PR) scores from 10 min (*p* < 0.001). Meaningful reduction in pain.	[97]
Prospective pilot	Germany	49	Completed	Stabilization or improvement of life quality 50% decrease in serum PSA level Decreased pain	[98]

## Data Availability

Not applicable.

## Abbreviations

Apple pectin (AP), Fiber pectin (FP), High methoxy pectins (HP), Low methoxy pectins (LP), Low-molecular-weight CP (LCP), Modified pectin (MP), Modified citrus pectin (Pect-MCP), Protein galectin 3 (Gal-3), Pomegranate fruit extract (PFE), Pectin extract (GW), PectaSol-C Modified Citrus Pectin (Pect-MCP), Pectin modified magnetic nano-particles (Fe_3_O_4_/Pectin/Au), Pectin oligosaccharide (POS), Pectin-capped gold nanoparticles (PEC-AuNPs), Pectin-guar gum-zinc oxide (PEC-GG-ZnO), Pectin mediated gold nanoparticles (p-GNPs), 5-FU loaded pec-tin-based nano-particles (5-FU-NPs), Zataria multiflora essential oil (ZEO).
